# Treatment-Limiting Decisions in Patients with Spontaneous Intracerebral Hemorrhage

**DOI:** 10.3390/medicina58080989

**Published:** 2022-07-25

**Authors:** Felix Lehmann, Matthias Schneider, Joshua D. Bernstock, Christian Bode, Valeri Borger, Stefan Felix Ehrentraut, Florian Gessler, Anna-Laura Potthoff, Christian Putensen, Lorena M. Schenk, Julian Zimmermann, Hartmut Vatter, Patrick Schuss, Alexis Hadjiathanasiou

**Affiliations:** 1 Department of Anesthesiology and Intensive Care, University Hospital Bonn, 53127 Bonn, Germany; christian.bode@ukbonn.de (C.B.); stefan.ehrentraut@ukbonn.de (S.F.E.); christian.putensen@ukbonn.de (C.P.); lorena_maria.schenk@ukbonn.de (L.M.S.); 2 Department of Neurosurgery, University Hospital Bonn, 53127 Bonn, Germany; matthias.schneider@ukbonn.de (M.S.); valeri.borger@ukbonn.de (V.B.); anna-laura.potthoff@ukbonn.de (A.-L.P.); hartmut.vatter@ukbonn.de (H.V.); patrick.schuss@ukb.de (P.S.); alexis.hadjiathanasiou@ukb.de (A.H.); 3 Department of Neurosurgery, Brigham and Women’s Hospital, Harvard Medical School, Boston, MA 02115, USA; jbernstock@partners.org; 4 Department of Neurosurgery, University Hospital Rostock, 18057 Rostock, Germany; florian.gessler@med.uni-rostock.de; 5 Department of Neurology, University Hospital Bonn, 53127 Bonn, Germany; julian.zimmermann@ukbonn.de; 6 Department of Neurosurgery, BG Klinikum Unfallkrankenhaus Berlin, 12683 Berlin, Germany

**Keywords:** intracerebral hemorrhage (ICH), treatment-limiting decisions (TLD), advanced directives, treatment futility

## Abstract

*Background and Objectives*: Treatment-limiting decisions (TLDs) are employed to actively withhold treatment/invasive interventions from patients in whom clinicians feel they would derive little to no benefit and/or suffer detrimental effects. Data regarding the employment of TLDs in patients with spontaneous intracerebral hemorrhage (ICH) remain sparse. Accordingly, this study sought to investigate both the prevalence of TLDs and factors driving TLDs in patients suffering from spontaneous ICH. *Materials and Methods:* This was a retrospective study of 249 consecutive patients with ICH treated from 2018–2019 at the Neurovascular Center of the University Hospital Bonn. Reasons deemed critical in the decision-making process with regard to TLD were ultimately extracted/examined via chart review of qualifying patients. *Results*: A total of 249 patients with ICH were included within the final analyses. During the time period examined, 49 patients (20%) had advanced directives in place, whereas in 53 patients (21%) consultation with relatives or acquaintances was employed before further treatment decisions. Overall, TLD ultimately manifested in 104 patients (42%). TLD was reached within 6 h after admission in 52 patients (50%). Congruent with severity of injury and expected outcomes, TLDs were more likely in patients with signs of cerebral herniation and an ICH score > 3 (*p* < 0.001). *Conclusions*: The present study examines details associated with TLDs in patients with spontaneous ICH. These data provide insight into key decisional processes and reinforce the need for further structured investigations in an effort to help guide patients and their families.

## 1. Introduction

Spontaneous intracerebral hemorrhage (ICH) is a common neurologic emergency associated with severe morbidity and mortality [1,2,3]. While maximal medical management, surgical interventions and/or invasive monitoring may be warranted, the prospect of survival and the ability to regain an acceptable quality of life is limited. As such, withholding/withdrawal of life-sustaining treatments is often broached in these clinical contexts [4]. Unsurprisingly, such decisions can be enormously disruptive for both patients and their families. In cases where key stakeholders (i.e., patients if able vs. family) agree that further interventions would in fact be futile, treatment-limiting decisions (TLDs) may ultimately be implemented [5]. Clinicians not only shoulder the medical responsibility for implementing such TLDs, but often face additional time constraints in decision-making and providing support/guidance to patients/family members. The burden that such situations provide for both family members and hospital staff might be mitigated, expedited and directed by advance directives of the patient’s will. However, not only ethical, moral views might vary considerably within the involved social milieu of an affected patient, but even the clinical implementation/execution of a TLD might attain a significant degree of heterogeneity [4,6]. Therefore, a further scientific exploration of this topic is essential in view of the increasing number of informed/educated patients and the growing complexity of intensive care capabilities.

There is only limited evidence on the factors that influence TLDs related to spontaneous ICH. Therefore, the present study aims to provide an insight into decision making regarding TLDs in a consecutive patient population at a university neurovascular center.

## 2. Materials and Methods

### 2.1. Patients

All consecutive patients with spontaneous non-traumatic ICH referred to the Neurovascular Center of the University Hospital Bonn over a 2-year period (i.e., from 2018–2019) were identified using an institutional database. Patients in whom ICH could also potentially have occurred as a result of an underlying cause (e.g., tumor, cavernoma, and/or aneurysm) were discarded from further analysis after application of a diagnostic sequential regimen [7]. After applying the inclusion/exclusion criteria, data was retrospectively obtained and included patient characteristics, location and volume of the ICH [8], neurological status on admission via the Glasgow Coma Scale (GCS), ICH score [1], existence of advance directives, as well as details with regard to any TLD that was ultimately made.

### 2.2. Treatment-Limiting Decisions

For each patient in whom a TLD was made, details were categorized as previously described by Robertsen et al. into the following categories: withholding surgery, withholding admission to intensive care unit (ICU), withholding organ support, any order not to resuscitate (DNR), any order not to escalate (DNE), withdrawal of intracranial pressure (ICP) specific treatments, and/or withdrawal of organ support [9]. The TLDs made were further classified into an additional three categories: only withholding life-sustaining treatment, only withdrawing life-sustaining treatment, and hybrid TLDs including both withholding and withdrawing. In addition, the location of patients for which each TLD was made was recorded: emergency room (ER), ICU, or ward. Regarding the timing of TLDs, three time points of interest were distinguished: <6 h, 6 to 24 h, >24 h after admission.

### 2.3. Statistics

SPSS (version 25, IBM Corp., Armonk, NY, USA) was utilized for data analyses. For comparison of continuous variables, the Mann-Whitney U test was employed given that the data were abnormally distributed. For parametric statistics, an unpaired t-test was used after testing for normality. Categorical variables were tested in contingency tables using Fisher’s exact test. The level of significance of results was set to *p* < 0.05.

## 3. Results

### 3.1. Patient Characteristics

249 patients with spontaneous non-traumatic ICH were admitted to the authors’ Neurovascular Center between 2018 and 2019. The median age was 76 years (interquartile range (IQR) 65–82) and 49% of the patients were male (121/249). The median ICH score of patients was 2 (IQR 1–3), with 48 patients (19%) presenting with an ICH score > 3 at the time of admission. In-hospital mortality was 39%. The decision to proceed with a TLD was made for 104 patients (42%).

### 3.2. Advanced Directives

Overall, 49/249 patients with ICH (20%) possessed an advance directive. In 53/249 patients with ICH (21%), any treatment decision was made in consultation with the family in accordance with the patient’s presumed wishes. In 10/249 patients (4%), the treating physician’s opinion was employed when a TLD was deemed necessary and the family was absent. Of note, in 137/249 patients with ICH (55%), no advance directives were available and/or no discussions with family members/caregivers regarding potential TLDs had unfolded prior to the examined hospitalization.

Of those patients who presented with advance directives, the median age was 82 years (IQR 77–88). This sub-cohort was significantly older than patients without advance directives, who presented with a median age of 74 years (IQR 62–80; *p* < 0.001). Further features of patient characteristics are listed in Table 1.

### 3.3. Treatment-Limiting Decisions

**Figure 1:**
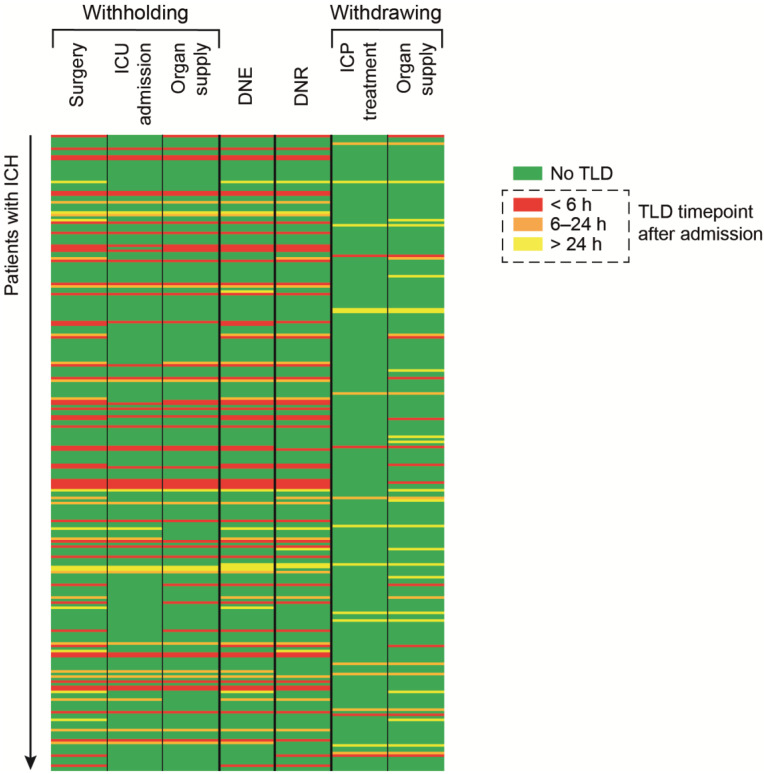
Illustrated synopsis of the TLD-making process, including its time-related context. ICH, intracerebral hemorrhage, ICU, intensive care unit, DNE, do-not-escalate, DNR, do-not-resuscitate, ICP, intracranial pressure, TLD, treatment-limiting decisions.

As per the above-mentioned 104 patients with ICH (42%), a TLD was deemed necessary at the time of admission or during the course of their hospitalization. TLDs were categorized as follows: only withholding TLDs in 58% (60/104), only withdrawing TLDs in 22% (23/104), and as hybrid TLDs in 20% (21/104). A detailed description of the TLDs is provided in both Figure 1 and Table 2.

TLDs were made in 23 patients during their stay in the ER (22%), in 46 patients after (multiple) discussions/consultations in the ICU (44%), and in 35 patients during their time on the general ward (34%). In 52 patients, TLD ensued within 6 h (50%), in 24 patients within the first 24 h (23%), whereas in 28 patients more than 24 h were required for a TLD to unfold (27%).

Patients for whom TLDs were made were significantly more likely to demonstrate signs of cerebral herniation at the time of admission (37%) as compared to those patients for whom a TLD was not made (2%; *p* < 0.001, OR 27.2, 95% CI 8.1–91.5). Patients with TLD were more often female as compared to patients in whom TLDs were not reached (63% vs. 43%; *p* = 0.001, OR 2.3, 1.4–3.9). ICH score was also associated with TLDs: 38% of patients in whom TLD was put into effect during treatment course exhibited an ICH score > 3 compared to 6% of patients with ICH score > 3 without TLD (*p* < 0.001, OR 10.7, 95% CI 4.7–24.2).

## 4. Discussion

Spontaneous intracerebral hemorrhage can have a wide variety of underlying causes [10,11,12,13,14] However, the common denominator is that the ICH is an unexpected and devastating event in the lives of affected patients due to the sudden bleeding event [15]. Due to this unpredictability, treating physicians also regularly encounter a problem in patients with ICH that is well known from other areas of intensive care/emergency medicine: an expression of the specific patient’s will is oftentimes not possible or not reliable due to the severity of the disease and/or neurological deficits [9,16]. In the case of intracerebral hemorrhage, however, drastic deficits are often to be expected (depending on the localization of the disabling hemorrhage), which either entail a need for nursing care or are even incompatible with an independently continued life [17]. In the case of disastrous prognostic situations, it is the ethical responsibility of the treating physicians to formulate an individual therapeutic goal. Even if this is difficult by consensus, according to national ethical guidelines, physicians are further obliged to elicit the patient’s presumed preference through (several) discussions with their relatives, friends, and caregivers. An advance directive, despite its sometimes vague wording, provides the treating physician with an impression of the patient’s own preferences in these situations.

Since a highly individual situation must be assumed for these cases, but the topic/conflict seems to be almost commonplace in neurological/neurosurgical intensive care units, academic discussions prove difficult in terms of their methodology/design. Nonetheless, complementing the scanty literature on this subject in patients with ICH, we reviewed the specificities of patients with ICH treated at our neurovascular center using distinguishable criteria as previously suggested [9].

Overall, it was observed that only 20% of patients had an advanced directive at the time of ICH on which basis the determination of an individual TLD was supported. This percentage might be explained by the abruptness of the bleeding event on one side and by the comorbid patient population (e.g., arterial hypertension, older age) on the other. However, a previous scenario-based trial concluded that physicians are not necessarily influenced by patient values in their prognostic assessment [18]. In the case of neurological/neurosurgical conditions, however, patient values are important due to the often permanent impairment resulting from these conditions. Patients with ICH may be incapable of decision making and their family/friends are then asked to officiate as advanced surrogate directives. However, family members often express distrust when confronted with TLD and repeated consulting throughout treatment is necessary [19]. In the present study, the presumed will of the patient with ICH was clarifiable with the aid of relatives in a further 21% of cases. Therefore, a reliable statement regarding the potential patient’s will was available for about half of the patients in the present 2-year study.

However, advanced directives (in writing) are often not available in emergency situations [20], especially if elicited through multiple consultations with relatives. This circumstance often leads to a shift of decision-making responsibilities into the emergency room. Here, once the diagnosis has been made, initial treatment-related decisions are initiated (intubation, conservative intracranial pressure therapy, catecholamine therapy, etc.). In the present series, TLD had occurred in 42% of patients with ICH. This is a high number compared with reports about patients with severe traumatic brain injury (17%), supporting the importance of the present study [9]. One-half of all TLDs were reached within the first six hours of hospital admission, making it worthwhile to consider the manner of the obtained TLD. Thus, TLDs might be distinguished between withholding and withdrawing further treatment [21]. The former suggests a straightforward decision-making process at the onset of (any) therapy, whereas, in the case of withdrawing, additional diagnostic tests and/or discussions appear to be necessary in order to identify more precise prognostic factors. In the present study, a large proportion of patients with ICH and TLD were assigned to withholding therapy (58%). Therefore, based on the (potential) patient’s will and the corresponding clinical findings, decisions were made at an early stage that seem to have prevented unnecessary/unwanted therapies/interventions. That patients with ICH and need for TLD presented in clinically more deteriorated condition (cerebral herniation, ICH score > 3) than patients without TLD emphasizes the importance and influence of medical judgment of the likelihood/severity of potential impairment. Nevertheless, especially in the acute clinical picture of ICH, there is often the problem that a rapid surgical intervention, even in unawareness of the patient’s presumed will, may be necessary [13,22,23]. According to the “hit hard and early” principle, a timely de-escalation in the sense of the patient’s will would then be possible in the short term after clarification of the further circumstances. Therefore, the integration of palliative/comfort care services into ICU workflow could be beneficial in the implementation of TLD and could prevent unnecessary interventions [24].

Several authors have already addressed the imponderability and unpredictability of end-of-life decision making in patients with spontaneous ICH [25,26]. The circumstance of self-fulfilling prophecy is frequently mentioned, since a withdrawal of intensive care support is very likely to lead to the (expected) poor outcome [25]. Nevertheless, decision-making is not based on purely medical characteristics/parameters. The very high proportion of individual ideas/wishes/hopes of the patients themselves and/or their social context can either support the medical assessment or often contradict it. For this reason, the present retrospective study has also attempted a time-related reconstruction of the decision-making process.

The present report describing institutional decision-making processes among patients with ICH is intended to provide a comparative dataset due to the lack of available evidence on patients with ICH. Furthermore, according to the view of the authors, it is also necessary to support the treating physician’s assessment as well as its consistent communication (to families and caregivers) in case of absent advanced directives in patients with severe and debilitating conditions (herein: ICH).

This study is limited by its retrospective nature and the fact that the complexities involved in TLD processes can only be reconstructed via chart review/documentation. Furthermore, full coverage of a defined period of time yields distinctly heterogeneous but real-world patient populations. However, this also combines patient groups that require different clinical handling. This should be taken into account when assessing the results of our study.

## 5. Conclusions

The present study explores the details of TLD making in patients with ICH at a major University center. The data presented provide some insight into key decisional processes and reinforce the need for further prospective investigations in an effort to help guide patients and their families.

## Figures and Tables

**Table 1:** Patients’ characteristics.

	Patients with ICH w/o TLD (*n* = 145)	Patients with ICH and TLD (*n* = 104)	
Median age (years, IQR)	72 (59–79)	80 (74–84)	*p* < 0.0001
Female sex	62 (43%)	66 (64%)	*p* = 0.001, OR 2.3, 95% CI 1.4–3.9
Anticoagulation/antiplatelet medication	66 (46%)	59 (57%)	*p* = 0.1
GCS ≥ 13	99 (68%)	23 (22%)	*p* < 0.0001, OR 7.6, 95% CI 4.2–13.5
Infratentorial ICH location	19 (13%)	18 (17%)	*p* = 0.4
ICH volume < 30 mL	103 (71%)	41 (39%)	*p* < 0.0001, OR 3.8, 95% CI 2.2–6.4
Presence of clinical signs of herniation at admission	3 (2%)	38 (37%)	*p* < 0.0001, OR 27.3, 95% CI 8.1–91.5
History of cancer	15 (10%)	15 (14%)	*p* = 0.3
Presence of IVH	45 (31%)	69 (66%)	*p* < 0.0001, OR 4.4, 95% CI 8.1–91.5
Known advanced directive	11 (8%)	38 (37%)	*p* < 0.0001, OR 7.0, 95% CI 3.4–14.6

ICH, intracerebral hemorrhage; w/o, without; TLD, treatment-limiting decision; IQR, interquartile range; GCS, Glasgow Coma Scale; IVH, intraventricular hemorrhage.

**Table 2:** Characteristics of TLDs in patients with ICH.

TLD	Patients with ICH (*n* = 249)
withholding surgery	79 (32%)
withholding access to ICU	53 (21%)
withholding organ support	58 (23%)
DNR	75 (30%)
DNE	73 (29%)
withdrawing ICP-targeted treatment	19 (8%)
withdrawing organ support	42 (17%)

DNE, do-not-escalate; DNR, do-not-resuscitate; ICH, intracerebral hemorrhage; ICP, intracerebral pressure; ICU, intensive care unit; *n*, number of patients; TLD, treatment-limiting decisions.

## Data Availability

Data is contained within the article.
